# Evaluation of analogs of mutacin 1140 in systemic and cutaneous methicillin-resistant *Staphylococcus aureus* infection models in mice

**DOI:** 10.3389/fmicb.2022.1067410

**Published:** 2022-12-14

**Authors:** Min Ju, Thushinari Joseph, Nopakorn Hansanant, Mengxin Geng, McKinley Williams, Andrew Cothrell, Andrew Riley Buhrow, Frank Austin, Leif Smith

**Affiliations:** ^1^Antimicrobial Division, Sano Chemicals Inc., Bryan, TX, United States; ^2^Department of Biology, Texas A&M University, College Station, TX, United States; ^3^College of Veterinary Medicine, Mississippi State University, Mississippi State, MS, United States

**Keywords:** methicillin-resistant *Staphylococcus aureus*, MRSA, antibacterial, mutacin 1140, efficacy, lantibiotics

## Abstract

Mutacin 1140 (Mu1140) is a potent antibiotic against Gram-positive bacteria, such as *Staphylococcus aureus*. The antibiotic is produced by the oral bacterium *Streptococcus mutans* and is a member of the epidermin family of type AI lantibiotics. The antibiotic exerts its inhibitory activity by binding to the cell wall precursor lipid II, blocking cell wall synthesis, and by disrupting bacterial membranes. In previous studies, the novel K2A and R13A analogs of Mu1140 have been identified to have superior pharmacokinetic properties compared to native Mu1140. In this study, the use of a combined formulation of the Mu1140 K2A and R13A analogs was shown to be more effective at treating MRSA bacteremia than the native Mu1140 or vancomycin. The analogs were also shown to be effective in treating an MRSA skin infection. The use of K2A and R13A analogs may provide a future alternative for treating serious Gram-positive bacterial infections. In a previous study, the Mu1140 analogs were shown to have significantly longer drug clearance times, leading to higher plasma concentrations over time. These properties warranted further testing to determine whether the analogs are effective for the treatment of systemic MRSA and acute skin infections. In this study, Mu1140 analogs were shown to be more effective than currently available treatments for systemic and skin MRSA infections. Further, the study clearly shows that the new analogs are superior to native Mu1140 for the treatment of a systemic MRSA infection. These findings support continued drug product development efforts using the K2A and R13A Mu1140 analogs, and that these analogs may ameliorate the outcome of serious bacterial infections.

## Introduction

Gram-positive bacterial infections, in particular multidrug-resistant bacteria, impose a serious burden on communities and the healthcare system. *Staphylococcus aureus* and other Gram-positive bacterial infections are often life-threatening diseases. Whether the infection is hospital or community acquired, healthcare systems are challenged with the financial burden and inadequate resources to treat these infections (CDC, 2021b). The CDC has reported that in 2017 methicillin-resistant *S. aureus* (MRSA) contributed to over 323,000 cases in hospitalized patients and that 10,600 of those patients died as a result of the infection (CDC, 2019). The overall cases from 2012 to 2017 had steadily decreased, but the National Acute Care Hospitals (ACHs) reported a significant increase in MRSA bacteremia between 2019 and 2020 (CDC, 2021a). The mortality rate associated with MRSA bacteremia has not improved with the antibiotics currently available (Burgin et al., 2022). The increasing tolerance of these infections to beta-lactams, aminoglycosides, and vancomycin is alarming. Even after several decades of use, vancomycin is still the first choice for the treatment of MRSA bacteremia. Daptomycin is noninferior to vancomycin and is primarily used as a salvage therapy when patients fail to respond positively to vancomycin (Liu et al., 2011; Burgin et al., 2022). Currently, there are no treatment options for MRSA bacteremia shown to be superior to vancomycin. It is critically important to develop new antibiotics that can be added to the dwindling arsenal of treatment options and possibly improve upon the available treatment options.

**Figure 1:**
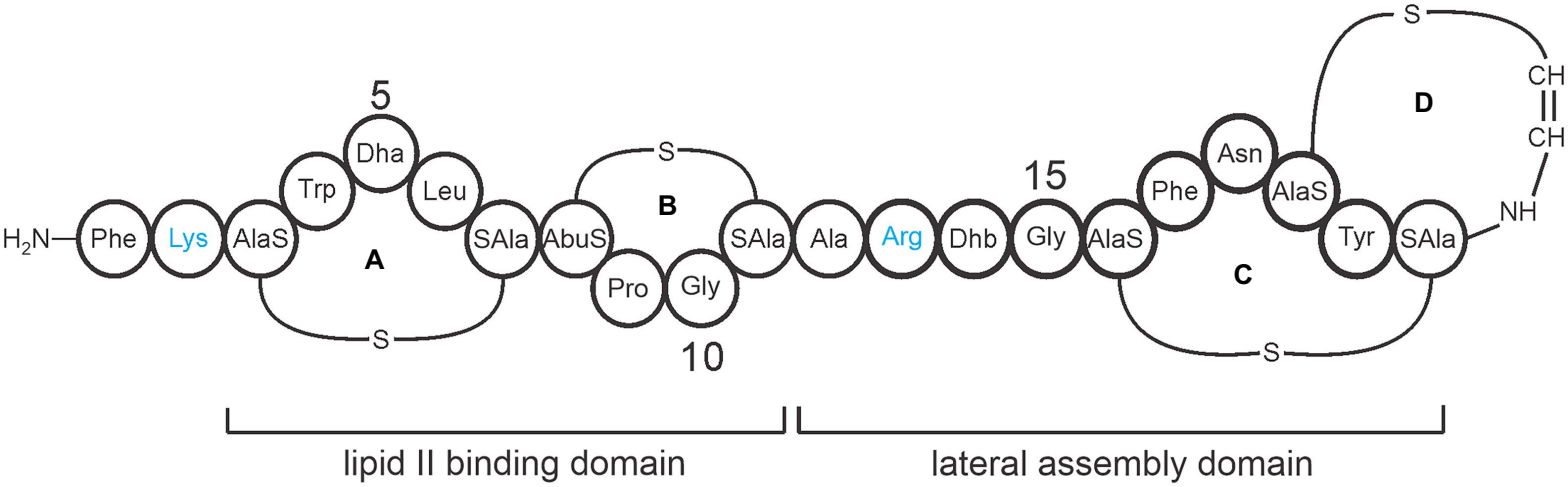
Representative covalent structure of Mu1140. The four lanthionine rings are labeled A, B, C, and D. The lipid II binding domain consists of rings A and B, while the lateral assembly domain consists of the hinge region (residues 12 to 15) and rings C and D. Protease-susceptible residues Lys and Arg are shown in blue and represent the sites for the alanine substitutions in the Mu1140 analogs. The amino acid abbreviations Dha is dehydroalanine and Dhb is dehydrobutyrine. The labels AlaS and SAla signifying the residues involved in the lanthionine ring formation, AlaS (Dha), AbuS (aminobutyric acid), and SAla (Cys).

A promising strategy for treating MRSA infections is to use ribosomally synthesized and post-translationally modified peptide (RiPP) antibiotics called lantibiotics. Specifically, engineered analogs of the lantibiotic mutacin 1140 (Mu1140) have been identified that have improved bioactivity, stability against proteases, and superior pharmacokinetic properties (Geng and Smith, 2018). Mu1140 is a peptide belonging to class I lantibiotics (Figure 1) and is naturally produced by a strain of the common oral bacterium, *Streptococcus mutans* JH1140. The antibiotic is ribosomally synthesized and undergoes enzymatic posttranslational modifications (PTM) leading to the formation of four lanthionine rings (Smith et al., 2000, 2003). The mechanism of action of Mu1140 is two-fold. Not only does it bind to the cell wall precursor, i.e., lipid II, to inhibit cell wall synthesis, the antibiotic also forms a uniform complex around the bacterial target that disrupts the bacterial membrane (Hasper et al., 2006; Smith et al., 2008).

A significant amount of work has gone into identifying effective structural analogs of Mu1140 for clinical development. A careful understanding of the structural elements that do not interfere with the antibiotic’s bioactivity or interfere with the antibiotic’s post-translational modifications was necessary to engineer the Mu1140 K2A and Mu1140 R13A analogs containing all of the PTM modifications (Escano et al., 2015; Geng et al., 2018; Geng and Smith, 2018). Interestingly, alanine substitutions within the core peptide would result in the loss of PTM modifications in unrelated regions of the peptide. This work suggested that the amino acid composition of the core peptide coordinates PTMs through the structural dynamics of the peptide, thus ensuring that all PTMs are completed prior to transport (Escano et al., 2015; Geng and Smith, 2018). Our group has previously shown that C-terminal ring formation is essential for transport and is the last PTM modification to form (Escano et al., 2015). Mu1140 analogs with changes in more than one amino acid residue did not show additive improvements in bioactivity and often resulted in a loss of product formation when the mutations were combined (Geng and Smith, 2018). Both the Mu1140 K2A and Mu1140 R13A analogs were identified to have higher antimicrobial activity against tested MRSA and *Streptococcus pneumoniae* strains, superior pharmacokinetic properties, and stability than native Mu1140 (Geng and Smith, 2018).

The pharmacokinetic parameters derived from a noncompartmental analysis (NCA) of the K2A and R13A analogs and native Mu1140 were evaluated (Geng et al., 2018). The K2A analog had the lowest total clearance (CL) and the highest area under the plasma drug concentration-time curve (AUC), while R13A had the highest volumes of distribution (Vss), the longest mean residence time (MRT), and half-life (t1/2). Since both of these analogs showed improvements in different pharmacokinetic properties, an equal mix of these analogs was used for treating a Gram-positive MRSA infection. In a previous study in mice, the combination of the K2A and R13A analogs (K2A:R13A, 1:1) was shown to be effective in treating a systemic MRSA infection (Geng et al., 2018). In this study, we further expanded the systemic MRSA infection study to compare the activity of the combined K2A:R13A analogs to native Mu1140 and vancomycin. Further, the current study evaluated the use of Mu1140 and its analogs to treat acute MRSA skin infections.

## Materials and methods

### Bacterial strains, growth conditions, chemicals, and animals

The two infectious methicillin resistant bacterial strains used in the study were *S. aureus* ATCC 33591 and *S. aureus* TCH1516. *S. mutans* JH1140, JH1140 K2A, and JH1140 R13A strains were used to produce Mu1140 and Mu1140 analogs. Growth conditions and purification procedures for each strain were previously described (Geng and Smith, 2018). Vancomycin hydrochloride was purchased from VWR (Radnor, PA). Fusidic acid, linezolid, and daptomycin were purchased from Sigma-Aldrich (St. Louis, MO).

The procedures used for preparing bacterial inoculum for each infection study were performed in a similar manner. A single colony from an overnight culture plate was transferred into fresh THyex broth and allowed to grow at 37°C in a shaking incubator at 280 rpm. The cultures were grown to an optical density at 600 nm (OD_600_) between 0.6–0.8. After the incubation, bacteria cells were harvested by centrifugation at 3,000 × g for 5 min and resuspended in either sterile PBS or saline. The resuspension of the cells was repeated two times to remove excess media components and the final inoculum was resuspended to the desired cell density. Inoculum cell density was first estimated by OD_600_ measurements and was subsequently verified in each study by determining the CFUs.

Equal numbers of 5-8-week-old male and female BALB/c mice (Envigo, Indianapolis, IN, USA) were used in all the animal studies. Rearing conditions and experimental procedures were approved by the institutional animal care and use committee of Texas A&M under the number 2021–0273.

### Bioactivity assays

The extraction and purification of Mu1140 and its two analogs K2A and R13A were performed as previously described (Geng and Smith, 2018). Preparation of the antimicrobial agent and bacterial inoculum for MICs was performed by following the method described by the Clinical and Laboratory Standards Institute (CLSI; M07-A8; CLSI, 2018) and as previously reported by our group (Chen et al., 2013; Escano et al., 2017; Geng and Smith, 2018). The bactericidal activity of K2A analog and vancomycin was determined using a time-kill study against *S. aureus* ATCC 33591 as previously reported with some minor modifications (Geng et al., 2018). A single colony from an overnight culture of *S. aureus* was inoculated in 10 ml of THyex broth (30 g/L Todd Hewitt broth, 3 g/L yeast extract). The inoculum was placed in a shaking incubator at 37°C and 280 rpm and grown to an OD_600_ of 0.6–0.8. The culture broth was then diluted to an OD_600_ of 0.132, after which a 20-fold dilution in fresh broth was performed resulting in a ~5 × 10^5^ CFU/ml cell density. Mu1140 and Mu1140 analogs were solubilized in dimethyl sulfoxide (DMSO) at a concentration of 1 mg/ml as the stock solution. An 8 ml aliquot of the inoculum was supplemented with K2A or vancomycin, resulting in a final concentration equivalent to 0.5×, 1×, and 2× MIC. The same volume of DMSO was added to all test samples including the no-drug control. The cultures were placed in a shaking incubator at 37°C and 200 rpm (Stuart orbital incubator SI500). A 100 μl sample from each culture was taken at each time point of 0, 0.25, 0.5, 0.75, 1, 1.5, 2, 4, 8, 18, and 24 h and was serially diluted in fresh medium before being spread on THyex agar plates (30 g/L Todd Hewitt broth, 3 g/L yeast extract, 15 g/L agar) for determining the CFUs. The plates were allowed to grow for 24 h at 37°C before the colony-forming units (CFUs) were counted. The CFU/ml for each time-point at each drug concentration was determined using at least two independent cultures. Plates having 30 to 300 colonies were used for CFU measurements.

### *In vivo* toxicity study

A single high i.v. dose (50 mg/kg) of the Mu1140 analogs K2A or R13A were administered *via* tail vein. Drug substance vehicle (pH = 3 citrate buffer with 1% Tween 80) was used as a negative control. Each of the three groups consisted of three male and three female mice. Mice were sacrificed approximately 24 h after administration. Blood was collected into BD Vacutainer™ blood collection tubes and transported on ice immediately to Texas A&M Veterinary Medical Diagnostic Laboratory (College Station, TX) for serum chemistry analysis. Parameters tested were total serum protein, albumin, calcium, phosphorous, glucose, BUN, creatinine, total bilirubin, ALP, AST(SGOT), ALT(SGPT), globulins, A/G ratio, GGT, amylase, cholesterol, sodium, potassium, Na/K ratio, and chloride. Animal carcasses were preserved in prefilled histology containers with 10% neutral buffered formalin (VWR®). Histological examination of heart, spleen, liver, kidney, thymus, duodenum, jejunum, ileum, colon, adrenal gland, pancreas, and testes (Male)/ovaries & uterus (Female) was performed by a board-certified pathologist at the College of Veterinary Medicine Diagnostic Laboratory at Mississippi State University.

### *In vivo* MRSA systemic infection model

Mu1140, combined analogs K2A and R13A (1:1), and vancomycin were tested for their ability to treat a systemic MRSA (*S. aureus* ATCC 33591) infection. The infection model was performed as previously reported (Geng et al., 2018) and the intravenous dose of K2A:R13A, Mu1140, and vancomycin were maintained at 10 mg/kg. Four groups of mice (*n* = 12 for the no-drug vehicle control and vancomycin groups, *n* = 6 for the Mu1140 wildtype and K2A:R13A groups) were infected *via* an i.p. injection of ~0.5 ml (28.6 μl/gram body weight) of an MRSA suspension containing ~6.2 × 10^8^ CFU in phosphate-buffered saline (PBS) at pH 7.4. At 1 h post-infection, three groups of mice were treated with a single i.v. dose, through the tail vein, of 10 mg/kg K2A:R13A analogs (1,1), 10 mg/kg Mu1140, or 10 mg/kg vancomycin. The vehicle (saline with 15% DMSO) served as the no-drug control group of mice. Each mouse was monitored every 4 h during the first 2 days post-infection (day 0 to day 2), and every 8 h from day 2 to day 5. The mice were sacrificed when they had a 15% weight loss or on the basis behaviors indicating pain and suffering (unresponsive or abnormal locomotion).

### Intravenous treatment of an *In vivo* MRSA acute skin infection

In this trial, Mu1140, the R13A and K2A analogs, and vancomycin were administered i.v. to test for their ability to treat an MRSA skin infection. Mice were acclimatized to the environment for at least 7 days before initiating experiments. After shaving the hair on the dorsum of the mice, a 50 μl suspension of *S. aureus* ATCC33591 in PBS, containing 1.5 × 10^7^ CFUs, was injected subcutaneously (subq). No bleeding was observed during all injections. Animals were observed for 48 h until open wounds developed. Mice were separated into four groups (*n* = 8 for the treatment groups and *n* = 6 for the control). The 10 mg/kg/day of vancomycin, 10 mg/kg/day of Mu1140, and 10 mg/kg/day of combined K2A:R13A analogs were administered i.v. twice daily for 5 days *via* tail vein. The last group received the no-drug vehicle control (PBS with 5% DMSO). Weights of mice were measured at least once per day, while behavior and the wounds were monitored multiple times per day. Mice were sacrificed approximately 24-h after the last i.v. treatment (Day 6; D6). Dermal tissues at the infected sites were sampled and homogenized in sterile PBS. The homogenates were serially diluted and plated on THyex agar for determining CFUs.

### Topical and subcutaneous treatment of *in vivo* MRSA acute skin infections

Trial 1: In this trial, the infection model was performed following methods that were previously reported (Kugelberg et al., 2005; Guo et al., 2013; Simonetti et al., 2020). Mu1140, K2A analog, R13A analog, combined K2A:R13A analogs, and fusidic acid were applied topically to a wound on the dorsum of mice. Seven groups (*n* = 6 mice/group) were acclimatized to the environment for at least 7 days before the start of the experiment. Once the hair on the dorsum of the mice was shaved, a 1 cm incision was made and a 50 μl suspension of *S. aureus* ATCC TCH1516 in PBS, containing 2 × 10^7^ CFUs, was applied directly to the incision. Mice were housed individually until inoculum dried at the incision site (Day (D) 0). Approximately 24 h later (D1), treatment was applied topically twice daily for 6 days. The treatment groups consisted of 10 mg/kg/day of Mu1140, 10 mg/kg/day of K2A analog, 10 mg/kg/day of R13A analog, 10 mg/kg/day of combined K2A:R13A analogs, 10 mg/kg/day of fusidic acid, and 5 mg/kg/day of combined K2A:R13A analogs. Another group received the no-drug vehicle control (0.6% noble agar in citrate buffer). Weights of mice were measured at least once per day, while behavior and the wounds were monitored multiple times per day. Mice were sacrificed 1 day after the last topical treatment (D7). Dermal tissues at the infected sites were sampled and homogenized in sterile PBS. The homogenates were serially diluted and plated on THyex agar for determining CFUs.

Trial 2: In this trial, the infection model was performed following methods that were previously reported (Guo et al., 2013; Dejani et al., 2016). Mu1140, combined K2A:R13A analogs, and linezolid were applied subq by a 30-gauge needle around the scab of the wound on the dorsum of mice. Mice were acclimatized to the environment for at least 7 days before initiating experiments. After shaving the hair on the dorsum of the mice, a 50 μl suspension of *S. aureus* ATCC33591 in PBS, containing 1.5 × 10^7^ CFUs, was injected subq (D0). As noted in trial 1, no bleeding was observed during all injections. Animals were observed for 48 h until open wounds developed. Mice were separated into four groups (*n* = 8 for treatment and no-drug vehicle control groups; D2). Treatment groups received a total of 25 mg/kg/day of Mu1140, 25 mg/kg/day of combined K2A:R13A analogs, and 75 mg/kg/day of linezolid. The last group received the no-drug vehicle control (sterile water with 20% DMSO). Treatments were administered three times per day to the mice through a 3-point subq injection around the scab with the needle pointed toward the center of the wound. Weights of mice were measured at least once per day, while behavior and the wound sites were monitored multiple times per day. Mice were sacrificed approximately 24-h after the last subq treatment (D6). Dermal tissues at the infected sites were sampled and homogenized in sterile PBS. The homogenates were serially diluted and plated on THyex agar for determining CFUs.

### Statistical analysis

All response criteria were analyzed using a one-way analysis of variance (ANOVA) model with a significance set at *α* = 0.05. Means were separated by student’s *t*-test. The Kaplan–Meier Curve with the log-rank test was used to estimate the survival function in the MRSA systemic infection model in mice. Statistical analysis and graphing were performed with JMP® (15.0.0, SAS Institute Inc., Cary, NC), Excel (Microsoft® Excel® for Microsoft 365 MSO Version 2,208), and R (4.2.1, the R Foundation for Statistical Computing, Vienna, Austria).

## Results

### *In vitro* antimicrobial activity

*Staphylococcus aureus* 33591 is a methicillin resistant strain (MRSA) that is commonly used in antimicrobial resistance and infectious disease studies and the MICs against *S. aureus* 33591 were determined for all antibiotics used in the current study. Native Mu1140, K2A, R13A, and the combined K2A:R13A analogs’ minimum inhibitory concentrations (MICs) were determined along with the MICs of vancomycin, fusidic acid, and linezolid (Table 1). Mu1140 and K2A analog had a four-fold lower MIC compared to vancomycin, while the R13A analog and the combined K2A:R13A analogs had an eight-fold lower MIC compared to vancomycin. Native Mu1140 and the K2A analog had a two-fold lower MIC compared to fusidic acid and linezolid, while the R13A analog and combined K2A:R13A analogs had a four-fold lower MIC than fusidic acid and linezolid. The K2A analog had the same MIC as native Mu1140, while the R13A analog had a two-fold lower MIC compared to Mu1140. The combined K2A:R13A analogs’ minimum inhibitory concentrations (MICs) were determined along with the MICs of vancomycin against a panel of clinical MRSA isolates (Table 2) and showed that the combined K2A:R13A analogs MICs ranged between 0.25 and 4 μg/ml, while vancomycin ranged from 0.5 to 4 μg/ml.

**Table 1:** MICs (μg/ml) of Mu1140 WT, Mu1140 K2A, Mu1140 R13A, and control agents against MRSA 33591 strain used in animal studies in broth.

Mu1140				Controls		
WT	K2A	R13A	KR (1:1)	Vancomycin	Fusidic acid	Linezolid
0.5	0.5	0.25	0.25	2	1	1

**Table 2:** MICs (μg/ml) of combined K2A:R13A analogs and vancomycin against a panel of clinical MRSA isolates in broth.

Strain of *Staphylococcus aureus*	Vancomycin (μg/ml)	K2A:R13A (1:1) (μg/ml)
33591	2	0.25
HFH30364	2	1
640	1	2
644	2	4
30476	2	4
631	2	2
CO-34	2	1
602	2	4
641	2	2
GA442	4	4
N315	2	1
USA300	2	1
CA347	2	1
616	2	4
31225	1	2
HFH30522	1	2
CA513	4	2
611	2	4
CA-409	2	2
31258	1	2
SAEH06	0.5	2

**Figure 2:**
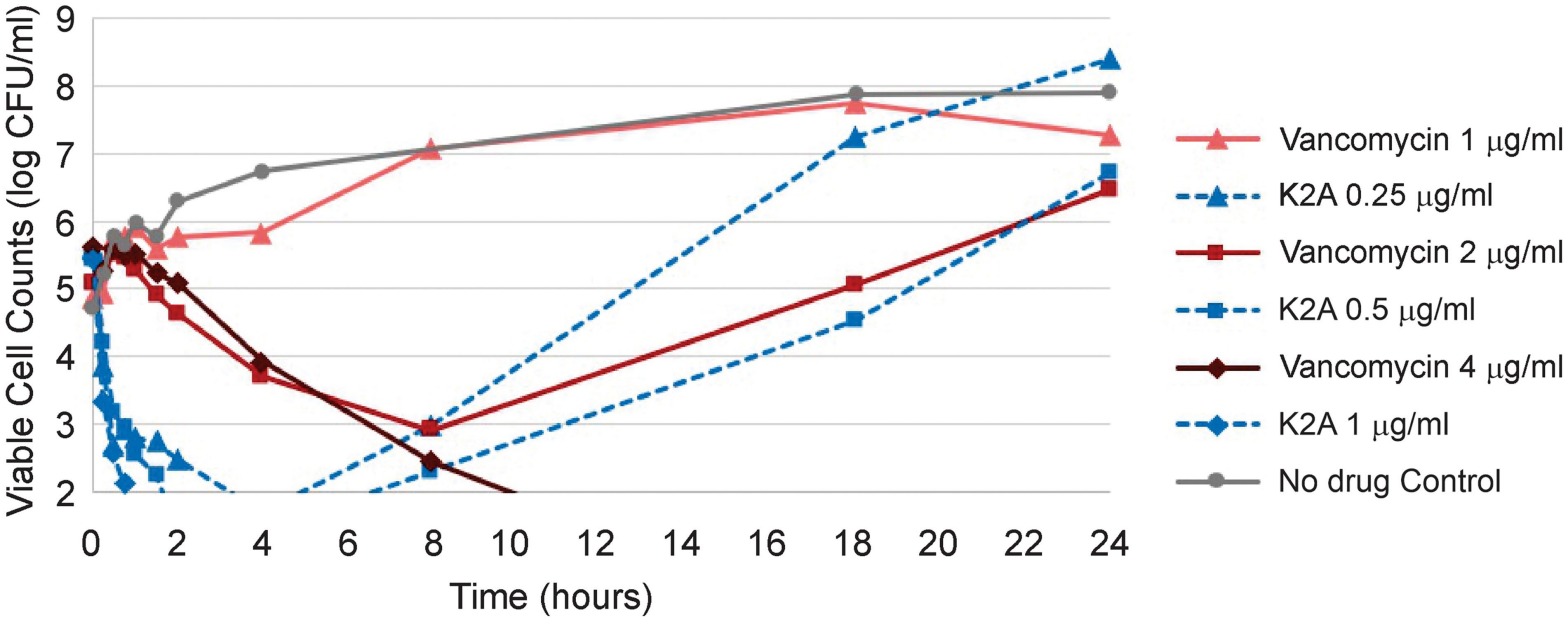
Kill kinetics of Mu1140 analog K2A compared to vancomycin. Half (0.5×) the MIC (1 μg/ml and 0.25 μg/ml for vancomycin and K2A, respectively), 1 × MIC (2 μg/ml and 0.5 μg/ml for vancomycin and K2A), and 2 × MIC (4 μg/ml and 1.0 μg/ml for vancomycin and K2A) were used to compare the differences in activity between K2A analog and vancomycin.

Mu1140, K2A, and R13A time-kill assays have been previously performed against *S. aureus* 33591. The K2A analog had the best killing effect in these studies and the viable cell numbers fell below the detection limit (<10^2^ CFUs/mL) after 2 h of exposure at 1× MIC. The time-kill assay was repeated in this study using K2A and vancomycin to better understand the differences in the rates of killing between the analog and vancomycin (Figure 2). The results showed that the K2A analog had a rapid bactericidal effect on the *S. aureus* 33591 culture compared to vancomycin. This effect was true for all the concentrations tested, 0.5× MIC, 1× MIC, and 2× MIC. At 1× MIC of K2A, the viable cell numbers fell below the detection limit by 2 h (> 3-log reduction) as previously reported. At 1× MIC of vancomycin, the treatment group had only a one-log reduction in viable cells by 2 h. At 0.5× MIC of vancomycin, there were negligible differences between the vehicle control and the treatment groups, while the 0.5× MIC of K2A treatment group had more than a 2-log reduction in viable cells. At 2× MIC, the K2A and vancomycin treatment groups had no viable cells at 18- and 24-h post-administration. The K2A dropped below the detection limit for viable cell counts after 1 h, while the 2× MIC treatment group of vancomycin took more than 8 h to drop the cell density below the detection limit.

### *In vivo* toxicity study

A single high intravenous (i.v.) dose (50 mg/kg) of K2A and R13A analogs was tested for toxicity in male and female mice. Blood samples were taken approximately 24 h after administration and the mice were euthanized and fixed for histopathological examination. The no-drug vehicle was used as the control group. Statistical analysis of variance found no difference among treatment groups on all parameters submitted in the serum chemistry tests (Supplementary Table S1). Histological examination of the heart, spleen, liver, kidney, thymus, duodenum, jejunum, ileum, colon, and testes (M)/ovaries (F) were all normal. No inflammation was observed in or around the renal tubules (Supplementary Table S2). The acute high dose toxicity study indicated that the K2A and R13A analogs were not toxic under the conditions evaluated in the study.

### MRSA systemic infection in mice

**Figure 3:**
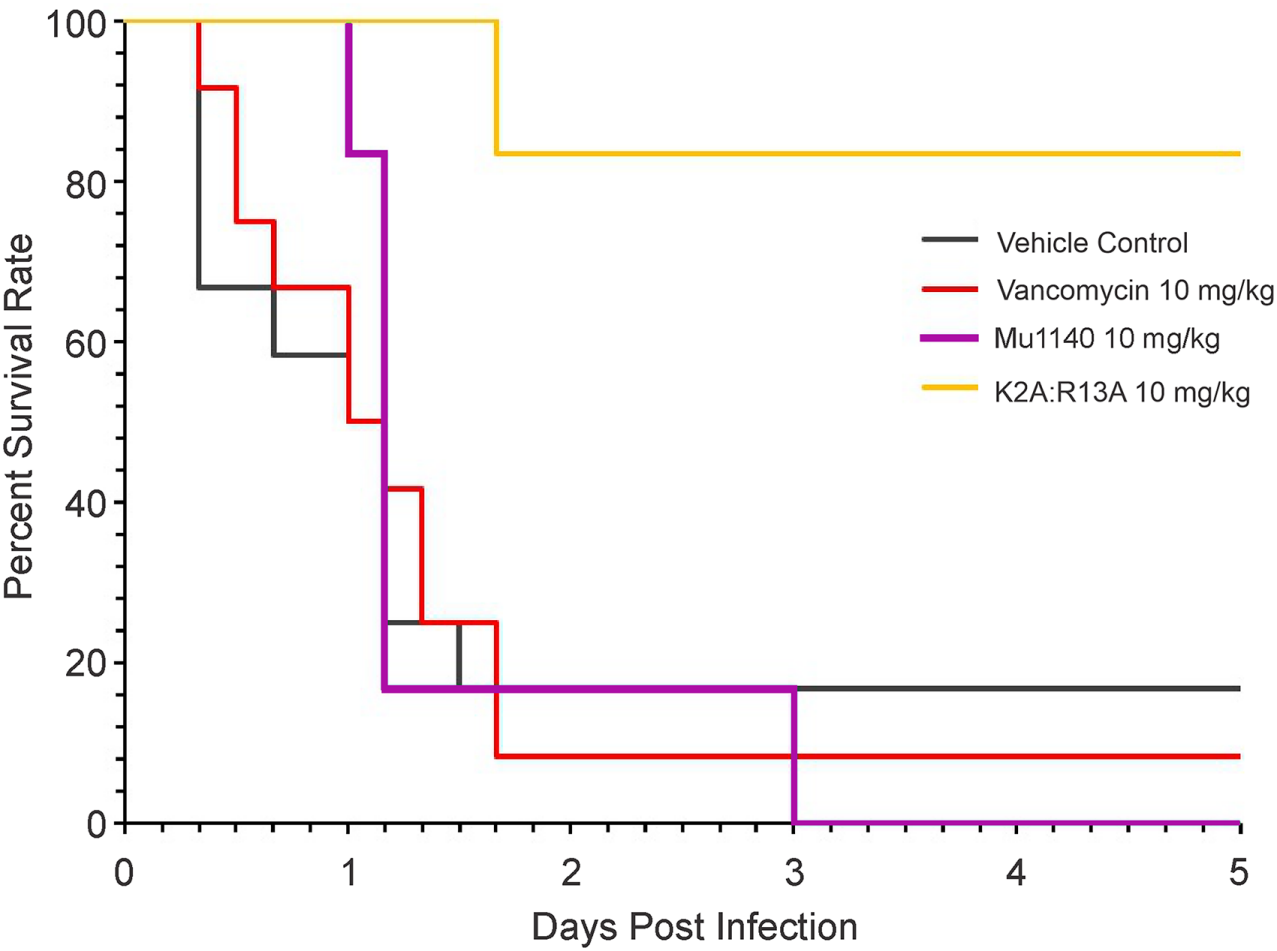
Survival curves of mice treated with vancomycin and mutacins *via* single-dose tail vein injection in a systemic MRSA infection. All treatments including the vehicle control were given to the animals in a sing-dose one-hour post-infection. Significance was observed among groups (log-rank test, value of *p* = 0.01). Pairwise comparisons using log-rank test showed statistical differences between K2A:R13A versus the control (value of *p* = 0.0115), vancomycin (value of *p* = 0.0065, and the Mu1140 (value of *p* = 0.0065). No difference was observed among the control, vancomycin, and Mu1140 groups (*n* = 12 for the vehicle control and vancomycin, *n* = 6 for the Mu1140 and K2A:R13A; all groups contained equal numbers of male and female mice).

Previously we reported that mice in an MRSA systemic infection treated with a single 10 mg/kg i.v. dose of combined Mu1140 analogs K2A and R13A (5 mg/kg of each analog) resulted in a 100% survival at day 5 and a single 2.5 mg/kg i.v. dose of the analogs (1.25 mg/kg of each analog) resulted in a 50% survival rate on day 5. The study was expanded to compare the efficacy of the combined K2A:R13A analogs to native Mu1140 and vancomycin (Figure 3). The combined analog treatment groups of the 10 mg/kg (K2A:R13A) provided superior protection against MRSA systemic infection compared to native Mu1140 and vancomycin. Significant differences were tested among the four treatment groups (Kaplan–Meier estimate of survival; log-rank test, *p* = 0.01; Peto & Peto modification of the Gehan-Wilcoxon test, *p* = 0.02). A single 10 mg/kg dose of K2A:R13A analogs (1:1) showed an 83.3% survival rate at day 5 and had a better subject survival probability than the other three groups (Pairwise comparisons using Log-Rank test, *p* = 0.0115 with the vehicle control, 0.0065 with the vancomycin, and 0.0065 with Mu1140) which all had lower than 20% survival rate at day 5. There was no statistical difference among the vehicle control, vancomycin, and Mu1140 groups. A 10 mg/kg i.v. dose of Mu1140 delayed the death compared to vehicle control and the vancomycin group, but there were no survivors by day 5.

### MRSA skin infection studies in mice

**Figure 4:**
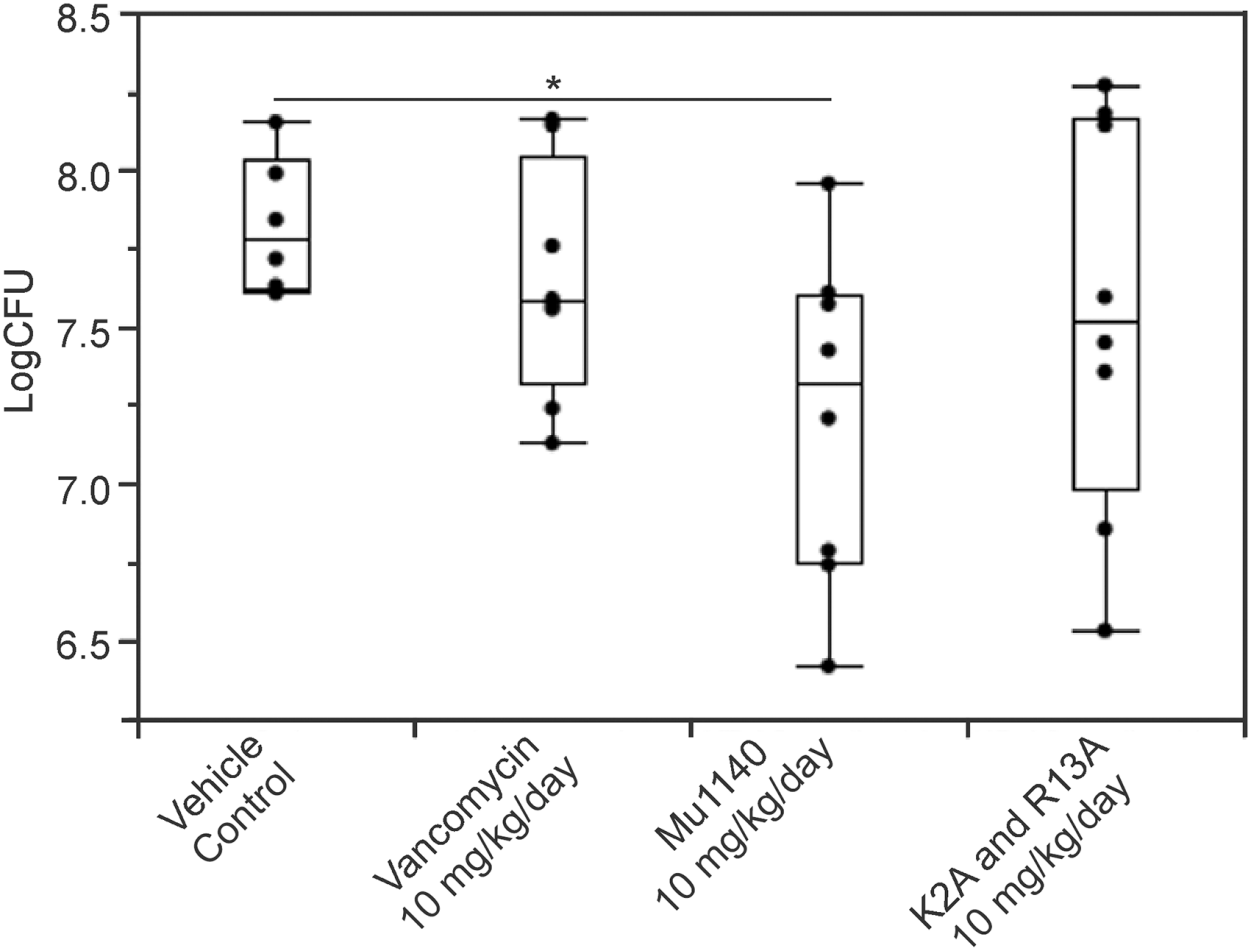
Bacteria load on the lesions of mice treated with vancomycin and mutacins *via* tail vein injection in a subcutaneous MRSA infection. All treatments including vehicle control were given to the animals 48 h post-infection and lasted for 5 days. Significance (*) was observed for the Mu1140 treatment group to the vehicle control group (mean difference = 0.61, Student’s *t*-test, Cohen’s *d* = 1.27, value of *p* = 0.0265; *n* = 6 for the vehicle control group, *n* = 8 for the other three groups, all groups contained equal numbers of male and female mice).

Mice were infected with MRSA via a subcutaneous (subq) injection on the dorsum and the mice were treated by i.v. administration for 5 days with Mu1140, K2A:R13A, and vancomycin at 10 mg/kg (Figure 4). A significantly lower bacteria count was observed in the 10 mg/kg Mu1140 treatment group compared to the no-drug control (log CFU = 7.21 versus 7.82, Student’s *t*-test, Cohen’s *d* = 1.27, value of *p* = 0.0265). The combined analog treatment group had a wide variability in bacterial load at the lesion site. Vancomycin used at 10 mg/kg i.v. dose did not have any significant effect on the bacterial load at the skin lesion site and the bacterial loads were similar to the vehicle control group. We tested daptomycin at a 25 mg/kg i.v. dose, using the same experimental conditions, and there was also no impact on the bacterial load at the lesion site (data not shown).

**Figure 5:**
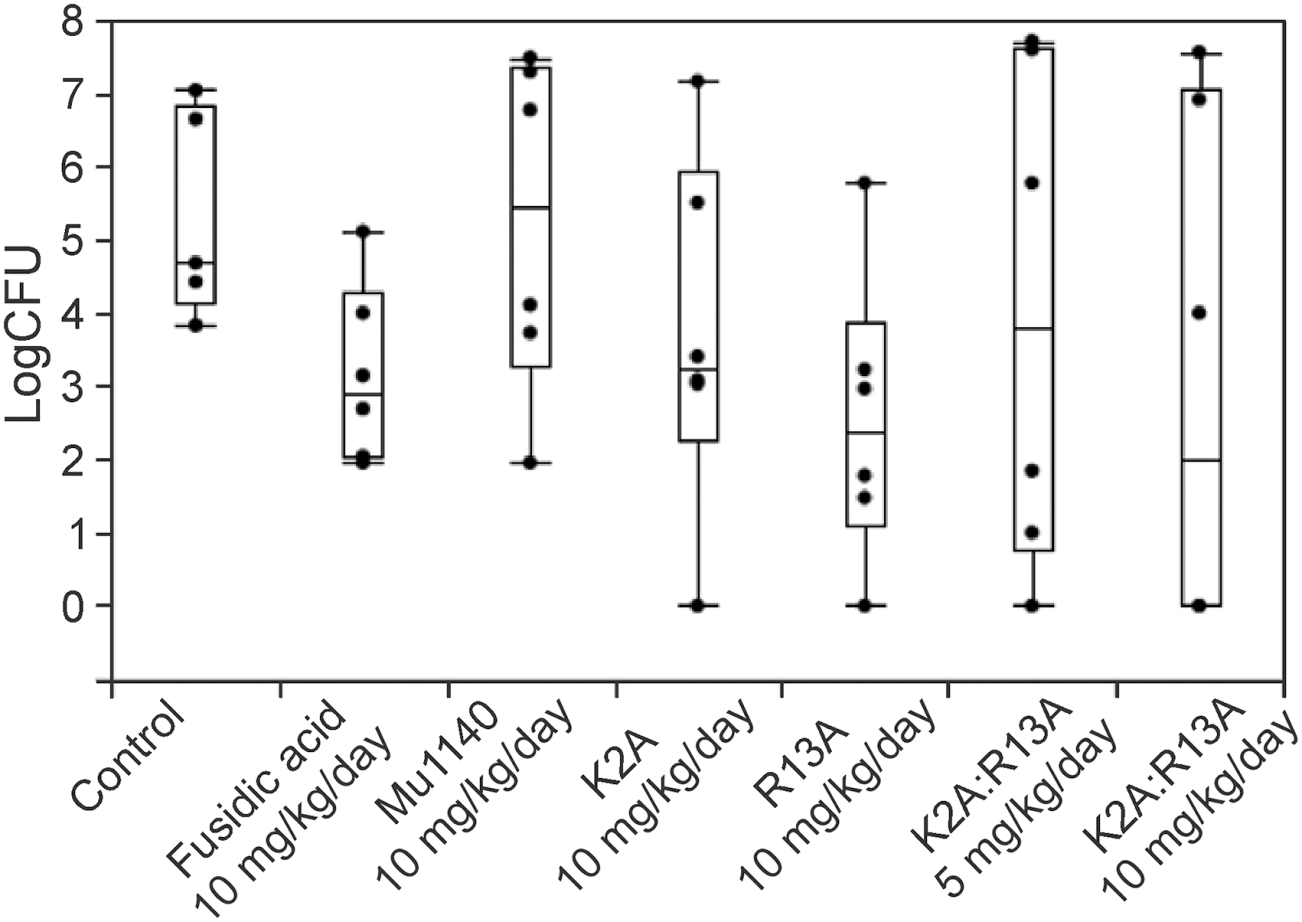
Bacteria load on the lesions of mice treated with fusidic acid and mutacins *via* topical application in a cut-wound MRSA skin infection. All treatments including vehicle control were given to the animals 24 h post-infection and lasted for 6 days. The model contributed to a wide range of variability in CFUs at the site of infection even for the no-drug vehicle control group. The mutacin analog treatment groups, along with fusidic acid group, had 1.34–2.79 logs lower average CFUs compared to the vehicle control group (mean log CFU = 5.33 for the control, 3.16 for fusidic acid, 5.23 for Mu1140 10 mg/kg/day, 3.70 for K2A 10 mg/kg/day, 2.54 for R13A 10 mg/kg/day, 3.99 for K2A:R13A 5 mg/kg/day, and 3.08 for K2A:R13A 10 mg/kg/day). No statistical difference was detected among treatment groups (*n* = 5 for the vehicle control group, *n* = 6 for all the other groups, all groups contained equal numbers of male and female mice except for the control group which lost one mouse due to a non-experimental reason).

Two topical application treatment studies were performed, and they differed in the introduction of the infection and by the application of the drugs. In the first study, an incision was made on the dorsum and a suspension of MRSA was applied to the wound and allowed to dry. Mu1140 and fusidic acid were formulated into gels and applied topically onto the wound for 6 days. The 10 mg/kg/day R13A analog group had the lowest mean bacterial load at the infected skin site with more than a 2-log reduction in bacterial load compared to vehicle control (Figure 5 and Table 3, mean difference = 2.79, Student’s *t*-test, Cohen’s *d* = 1.11, value of *p* = 0.0754). The 10 mg/kg/day combined K2A:R13A analog group and the 10 mg/kg/day fusidic acid treatment group had 2.25 and 2.17 log reductions in bacterial load compared to the vehicle control group (Student’s *t*-test, Cohen’s *d* = 0.89 and 0.86, value of *p* = 0.15 and 0.16). The 10 mg/kg/day K2A and the 5 mg/kg/day combined K2A:R13A analog treatment groups had 1.63 and 1.34 log reduction in bacterial load compared to the vehicle control group (Student’s *t*-test, Cohen’s *d* = 0.65 and 0.53, value of *p* = 0.29 and 0.39). The 10 mg/kg/day Mu1140 group had a similar mean bacterial load as the vehicle control group. The differences in bacterial load at the site of infection in all experimental groups were not statistically significant, due to the wide variability in the number of recovered bacteria at the site of infection. For instance, the control group had a 3-log variation in bacterial load at the site of infection. However, the bacterial load reduction between R13A and vehicle control group, and between R13A and native Mu1140 was noticeable (mean differences of 2.79 and 2.69, Student’s *t*-test, Cohen’s *d* = 1.11 and 1.07, *p*-value of 0.0754 and 0.0723, respectively).

**Table 3:** Group means (±SE) of log CFU/infection site of the experimental animals being treated by fusidic acid and mutacins *via* topical application in an incision MRSA skin infection model.

Treatment groups						
Vehicle control	Fusidic acid 10 mg/kg/day	Mu1140 10 mg/kg/day	K2A 10 mg/kg/day	R13A 10 mg/kg/day	K2A:R13A 5 mg/kg/day	K2A:R13A 10 mg/kg/day
5.33 ± 0.64	3.16 ± 0.50	5.23 ± 0.93	3.70 ± 1.00	2.54 ± 0.80	3.99 ± 1.41	3.08 ± 1.46

All treatments including the vehicle control were given to the animals 24 h post-infection and lasted for 6 days (*n* = 5 for the vehicle control group, *n* = 6 for all the other groups).

**Figure 6:**
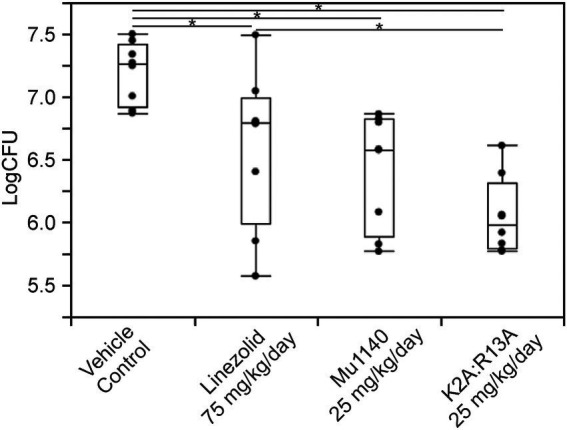
Bacteria load on the lesions of mice treated with linezolid and mutacins *via* subcutaneous injections in a subcutaneous MRSA skin infection. All treatments including vehicle control were given to the animals 48 h post-infection and lasted for 3 days. Significances (*) were observed between K2A:R13A and vehicle control (mean difference = 1.14, Cohen’s *d* = 2.64, value of *p* < 0.0001), Mu1140 and vehicle control (mean difference = 0.78, Cohen’s *d* = 1.80, value of *p* = 0.0012), linezolid and vehicle control (mean difference = 0.60, Cohen’s *d* = 1.39, value of *p* = 0.0097), and also between K2A:R13A and linezolid (mean difference = 0.54, Cohen’s *d* = 1.25, value of *p* = 0.0186). All groups had eight mice (*n* = 8) and contained equal numbers of male and female mice.

In the second topical administration study, the introduction of the bacterial infection and the application of the drug were refined to reduce the previously observed variability of bacterial load at the site of infection in each of the treatment groups. A suspension of MRSA was applied to the dorsum of mice by a subq injection. Open wounds were observed in all mice within 24–48 h post-infection. The vehicle control, 75 mg/kg/day linezolid, 25 mg/kg/day Mu1140, and 25 mg/kg/day K2A:R13A were administered *via* subq injection for three days. In order to promote a uniform application of each treatment to the wound and to ensure the drug was penetrating the hard scab, multi-point subq injections were used. As shown in Figure 6, significant reductions in bacterial loads at the skin infection site were observed among groups (ANOVA, Eta Squared = 0.51, value of *p* = 0.0001). All treatment groups were statistically significant compared to the no-drug vehicle control group (mean difference = 0.60–1.14; Student’s *t*-test; Cohen’s *d* = 1.39 for linezolid, 1.80 for Mu1140, 2.64 for K2A:R13A; value of *p* = 0.0097 for linezolid, 0.0012 for Mu1140, and < 0.0001 for K2A:R13A). The combined K2A:R13A analog treatment group also had significantly less bacterial burden than the linezolid group (mean difference = 0.54, Student’s *t*-test, Cohen’s *d* = 1.25, value of *p* = 0.0186) and performed the best at reducing the bacterial load at the infection site (>1-log reduction to the control group). No statistical differences were observed between Mu1140 and linezolid treated groups. In addition to a decrease in the bacteria load at the sites of infection for the antibiotic treatment groups, there were notable differences in the appearance of the wounds. Signs of wound healing such as reduction in redness, swelling, and wound size, were more apparent in all antibiotic treatment groups by day 3.

## Discussion

The pipeline for new antibiotics has modestly improved over the last 5 years (McCall, 2020; Iskandar et al., 2022), but the discovery and the development of new antibiotics with novel mechanisms of action compared to those already in use in the clinic is still lacking. Existing clinical antibiotics or therapies cannot effectively control multi-drug-resistance pathogens. Novel approaches are needed to alleviate the current healthcare problem. In a 2018 study, multilayered carbon nanotubes showed potential synergistic effect with methicillin when treating a multi-drug-resistant *Pseudomonas aeruginosa* infection (Mousavi et al., 2018). In a recent study, a co-expression network of genes involved in cell wall synthesis following exposure to penicillin in an *Escherichia coli* resistance strain has provided new targets for drug development (Shiri et al., 2020). Without the continued efforts made by academic research labs, the availability of useful approaches to mitigate human susceptibility to infections caused by drug-resistant bacteria will likely remain limited. Resistance or tolerance to commonly used antibiotics, such as vancomycin and β-lactam antibiotics, has restricted the treatment options against MRSA and other Gram-positive infections (Cázares-Domínguez et al., 2015; Burgin et al., 2022; Iskandar et al., 2022). The K2A and R13A analogs are both resistant to trypsin digestion and offer improved pharmacokinetic profiles compared to native Mu1140 (Geng and Smith, 2018). Both analogs had higher AUCs, and the K2A analog provided the highest peak plasma concentration, whereas the R13A analog had a significantly longer half-life. An equal combination of K2A and R13A was tested, given that the K2A would yield superior short-term bacterial clearance and the R13A would remain in plasma for prolonged effects. Previously reported *in vitro* and *in vivo* activity of the combined analogs K2A:R13A warranted further investigation into *in vivo* MRSA infection models.

Mu1140 and the K2A and R13A analogs were shown to have superior *in vitro* activity and kill-kinetics against MRSA 33591 compared to vancomycin, the preferred clinically used antibiotic for the treatment of serious MRSA infections (Table 1; Figure 2). Our lab has previously published data showing that the combined analogs of Mu1140 were effective in treating a systemic MRSA infection in a murine model (Geng et al., 2018). The previous results have been expanded in this study to show that the analogs of Mu1140 were more effective in treating a systemic MRSA infection in mice than native Mu1140 and vancomycin. Given the effectiveness of these analogs in the systemic infection study, the use of Mu1140 and the K2A and R13A analogs were tested against a serious MRSA skin infections and have been shown to be an effective treatment.

In the MRSA systemic infection model, a 10 mg/kg i.v. dose of K2A:R13A resulted in 83.3% survival of mice on day 5. At 12 h post-infection, the vehicle control group had 66.7% survival and the vancomycin group had 75.0% survival while Mu1140 and K2A:R13A treatment groups had a 100% survival rate. At 24 h post-infection, control and vancomycin both had 50% survival while Mu1140 had 83.3% survival and the K2A:R13A group had a 100% survival rate. Interestingly, the mutacin analogs group only showed death on day two post-infection and the remaining mice survived until day five. Treatment with K2A:R13A resulted in higher survival compared to native Mu1140 presumably due to the improved pharmacokinetic properties of the K2A and R13A analogs (Geng et al., 2018). By day 5, mice treated with 10 mg/kg vancomycin or 10 mg/kg Mu1140 showed no difference in survival probability with the control group nor between themselves. However, despite no survival on day 5, the Mu1140 treatment group still showed better protection during the early stages of infection than vancomycin. Given that vancomycin is the current treatment option for systemic MRSA, the results significantly highlight the superior activity of the combined analogs K2A:R13A to vancomycin at a 10 mg/kg dose in the murine systemic infection model. These observations support the need to further preclinical studies using K2A:R13, in particular pharmacotoxicity studies aimed at optimizing the i.v. dosing regimen of the Mu1140 analogs and preclinical safety studies.

The effectiveness of the K2A:R13A may be due to the rapid antibacterial activity of Mu1140 and its analogs. In the time-kill assay, the K2A analog at 2 × -MIC demonstrated a reduction in CFUs by more than 3-logs within 30 min with no recovery of bacterial culture over 24 h (Figure 2). The K2A analog at 0.5× and 1 × -MIC had similar levels of reduction in CFUs although bacterial load started to recover by 8 h onwards. In comparison, vancomycin at 1× and 2 × -MIC needed 8 hours for a 2-log reduction in CFUs. Rapid antibacterial activity is important for improved therapeutic outcomes during a systemic infection by reducing the bacterial load circulating throughout the body and minimizing the progression of the infection (Finberg et al., 2004). The relatively slow effect of vancomycin would likely allow for the infection to establish in other tissues, while the combined analogs of Mu1140 would prevent this dissemination. Possibly, the combined analogs of Mu1140 could be used in combination with other conventional antibiotics given its ability to rapidly reduce the bacterial load and that this could improve the therapeutic outcome of systemic MRSA infections. Altogether, the data suggest that i.v. administration of the K2A:R13A analogs may be a superior therapeutic option for systemic MRSA infections compared to vancomycin.

Given the effectiveness of K2A:R13A in treating systemic MRSA infection, the analog combination as well as Mu1140 was assessed in treating MRSA skin infections. Three different trials were conducted with different treatment options: i.v. administration, topical application, and subq administration. In the first study, treatment was administered *via* i.v. injections. In this study, Mu1140, but not the combined K2A:R13A analogs, showed a statistically significant reduction in CFUs at the site of infection. Treatment with 10 mg/kg/day i.v. dose of vancomycin also did not show any statistical significance to vehicle control. It was unexpected that the combined K2A:R13A analog did not show statistical improvements in bacterial load in this study, as the combination of these analogs was shown to have similar *in vitro* activity and superior pharmacokinetic properties (Geng et al., 2018). Given the rapid clearance of Mu1140 from blood as compared to the K2A and R13A analogs (Geng et al., 2018), possibly Mu1140 is more readily available at these superficial sites than the combined analogs as it is being cleared from the bloodstream. Nevertheless, the reduction in CFU by Mu1140 shows it may be a promising candidate in comparison to vancomycin as an i.v. administered skin infection treatment option.

The previously observed efficacy of K2A:R13A in systemic infection and its ineffectiveness in treating the skin infection made us consider alternative administration routes. The second study used topical application to treat skin infection, and the activity of Mu1140 and the combined K2A:R13A analogs were compared to the activity of fusidic acid. Fusidic acid has been used topically for decades and is often used in combination with other antibiotics (Figure 5; Dobie and Gray, 2004; Hajikhani et al., 2021). In this trial, mice were infected by having a 1 cm incision followed by the application of a bacterial suspension at 2 × 10^7^ CFUs. The study failed to show any significance, and this is likely due to the wide variation in CFUs at the site of infection. The control group mice varied by 3-logs and several of the treatment groups also had a wide variation in CFUs for each mouse. These observations are likely attributed to the failure of the infection model and not the drugs themselves, especially given that the means of the antibiotic treated groups were all around 2-logs lower than the control group (Table 3). Another potential issue with the topical application of the antibiotics in this MRSA model was likely the formation of a hard scab at the infection site, which would vary the penetration of the antibiotic leading to inconsistent treatments.

In the third study, the MRSA skin infection was established by using the subq injection method, given that the CFUs in the previous i.v. treatment model was more consistent between the mice. Repeating the MRSA skin infection by establishing infection with a subq inoculum and subq administration of the antibiotics at the site of infection reduced mouse to mouse variability in infection and drug treatment (Figure 6). Linezolid at 75 mg/kg/day had a 0.6-log reduction in CFUs (Cohen’s *d* of 1.39, value of *p* of 0.0097). Mu1140 and K2A:R13A at 25 mg/kg/day showed a 0.78-log (Cohen’s *d* of 1.80, value of *p* of 0.0012) and 1.14-log (Cohen’s *d* of 2.64, value of *p* of 0.0001) reduction in CFUs, respectively. The combined K2A:R13A analogs were shown to be statistically superior to linezolid at reducing CFUs at the site of infection (Cohen’s *d* of 1.25, value of *p* of 0.0186), but the combined analogs did not show superiority over native Mu1140. Future studies will need to be done to better understand the treatment duration, dosing regimen, and drug formulations for the Mu1140 and the combined K2A:R13A analogs.

## Conclusion

The *in vivo* systemic MRSA efficacy study supports the observed improvements in the pharmacokinetic properties of the K2A:R13A analogs and that these improvements do improve their availability and activity in the bloodstream. However, the improvements in the pharmacokinetic parameters (AUC, half-life, and peak plasma concentration) of the combined K2A:R13A analogs did not clearly show any superiority over native Mu1140 for treating an MRSA skin infection following i.v. administration. The combined analogs were shown to be effective in treating an MRSA skin infection following subq administration. Interestingly, Mu1140 and the combined analogs did repeatedly demonstrate statistically significant activity compared to conventional treatment options. Furthermore, acute dose toxicity studies indicated no behavioral or organ-specific toxicity by K2A and R13A even at an i.v. dose 5-times higher than what was used in the systemic MRSA infection study. Overall, the study strongly supports continued efforts toward the development of a novel drug using Mu1140 and the K2A:R13A analogs for treating serious Gram-positive bacterial infections.

## Data availability statement

The raw data supporting the conclusions of this article will be made available by the authors, without undue reservation.

## Ethics statement

The animal study was reviewed and approved by Texas A&M University IACUC 2021-0273—"Pharmacokinetic and efficacy studies of natural or structurally modified antimicrobial products isolated from bacteria (2021)."

## Author contributions

MJ, TJ, NH, MG, MW, AC, AB, FA, and LS either helped plan or perform experiments. MJ, TJ, NH, MG, MW, AC, AB, FA, and LS helped analyze the data. MJ, MG, and LS supervised the study and designed the experiments. MJ and LS drafted the manuscript. All authors contributed to the article and approved the submitted version.

## Funding

This study was funded by National Institute of Allergy and Infectious Diseases (1R41AI149803-01A1).

## Conflict of interest

LS and FA are board members of Sano Chemicals Inc. Sano Chemicals is actively developing analogs of mutacin 1140 for the treatment of serious bacterial infections. MJ, AB, and AC worked for Sano Chemicals as a research scientist.

The remaining authors declare that the research was conducted in the absence of any commercial or financial relationships that could be construed as a potential conflict of interest.

## Publisher’s note

All claims expressed in this article are solely those of the authors and do not necessarily represent those of their affiliated organizations, or those of the publisher, the editors and the reviewers. Any product that may be evaluated in this article, or claim that may be made by its manufacturer, is not guaranteed or endorsed by the publisher.
