# Supplementary material for: Evaluation of analogs of mutacin 1140 in systemic and cutaneous methicillin-resistant Staphylococcus aureus infection models in mice
Source: Front Microbiol. 2022 Dec 14;13:1067410. doi: 10.3389/fmicb.2022.1067410 (PMC9794991; doi:10.3389/fmicb.2022.1067410)
Supplement: Supplementary file 1 [file Table_1.DOCX]

Supplementary Table 1. Serum chemistry profile (automated photometry-small animal) of experimental mice after receiving a high dose of K2A or R13A in an *in vivo* toxicity study *

| Serum Chemistry | K2A  50 mg/kg | K2A  50 mg/kg | K2A  50 mg/kg | R13A  50 mg/kg | R13A  50 mg/kg | R13A  50 mg/kg | Vehicle Control | Vehicle Control | Vehicle Control | *Prob>F (One-way ANOVA)* | *Eta Squared* | *Std Error* |
| --- | --- | --- | --- | --- | --- | --- | --- | --- | --- | --- | --- | --- |
| Total serum protein, g/dL | 5 | 4.6 | 4.8 | 4.8 | 5.2 | 5.2 | 4.4 | 5.2 | 5.1 | *0.5902* | *0.161* | *0.177* |
| Albumin, g/dL | 3.2 | 2.4 | 2.7 | 3 | 3.1 | 3.1 | 2.7 | 3 | 3 | *0.4113* | *0.256* | *0.148* |
| Calcium, mg/dL | 9.4 | 9.3 | 9.2 | 9 | 9.2 | 9.2 | 8.3 | 9.3 | 9.3 | *0.5326* | *0.189* | *0.199* |
| Phosphorus, mg/dL | 8.9 | 7 | 7.8 | 7.1 | 7.6 | 7.6 | 31.5 | 7.9 | 7.1 | *0.4362* | *0.242* | *4.633* |
| Glucose, mg/dL | 131 | 117 | 154 | 162 | 165 | 185 | 129 | 184 | 195 | *0.1902* | *0.425* | *13.967* |
| BUN, mg/dL | 34 | 49 | 32 | 22 | 25 | 24 | >130 | 24 | 28 | *NA* | *NA* | *NA* |
| Creatinine, mg/dL | 0.2 | 0.3 | 0.2 | < 0.2 | 0.2 | <0.2 | 4.9 | 0.2 | 0.2 | *NA* | *NA* | *NA* |
| Total Bilirubin, mg/dL | 0.2 | 0.1 | 0.1 | 0.2 | 0.2 | 0.2 | 0.4 | 0.2 | 0.2 | *0.1715* | *0.444* | *0.043* |
| ALP, U/L | 161 | 100 | 118 | 133 | 141 | 180 | 139 | 171 | 156 | *0.3675* | *0.284* | *14.418* |
| AST (SGOT), U/L | 156 | 328 | 264 | 318 | 171 | 241 | 498 | 222 | 171 | *0.8418* | *0.056* | *69.853* |
| ALT(SGPT), U/L | 49 | 223 | 62 | 63 | 45 | 52 | 69 | 39 | 70 | *0.443* | *0.238* | *32.976* |
| Globulins, g/dL | 1.8 | 2.2 | 2.1 | 1.8 | 2.1 | 2.1 | 1.7 | 2.2 | 2.1 | *0.9771* | *0.008* | *0.126* |
| A/G Ratio | 1.8 | 1.1 | 1.3 | 1.7 | 1.5 | 1.5 | 1.6 | 1.4 | 1.4 | *0.6844* | *0.119* | *0.132* |
| GGT, U/L | <3 | <3 | <3 | <3 | <3 | <3 | 7 | <3 | <3 | *NA* | *NA* | *NA* |
| Amylase, U/L | 698 | 454 | 426 | 462 | 522 | 553 | 597 | 738 | 617 | *0.2562* | *0.365* | *58.066* |
| Cholesterol, mg/dL | 100 | 137 | 149 | 105 | 139 | 143 | 83 | 135 | 123 | *0.7009* | *0.112* | *14.258* |
| Sodium, mEq/L | 153 | 154 | 153 | 149 | 153 | 152 | 146 | 152 | 152 | *0.2924* | *0.336* | *1.361* |
| Potassium, mEq/L | 6.2 | 6.7 | 7.2 | 7.7 | 7.2 | 6.9 | >10.0 | 7.2 | 6.9 | *NA* | *NA* | *NA* |
| Na/K Ratio | 24.7 | 23 | 21.3 | 19.4 | 21.3 | 22 | 14.6 | 21.1 | 22 | *0.2911* | *0.337* | *1.528* |
| Chloride, mEq/L | 112 | 110 | 110 | 113 | 111 | 112 | 113 | 110 | 109 | *0.4921* | *0.211* | *0.861* |

* – n=3 for all the dependent variables of each group

Std Error – from pooled standard deviation

NA – not applicable

Supplementary Table 2. Pathology report of experimental mice in an in vivo toxicity study *

| Treatment | Sex | Observations |
| --- | --- | --- |
| K2A 50 mg/kg | F | Heart, Spleen, Liver, Kidney, Thymus, Duodenum, Jejunum, Ileum, Colon, Uterus, Adrenal, and Pancreas are normal. The lung had pulmonary hemorrhage and mild fibrin exudation attributed to peri-mortem euthanasia. There was a suppurative vaginitis present. |
| K2A 50 mg/kg | F | Spleen, Liver, Kidney, Adrenal, Thymus, Duodenum, Jejunum, Ileum, Colon, Stomach, Ovary, Uterus, Cervix, and Vagina are normal. The Heart had moderate Atrial mineralization. The lung had pulmonary hemorrhage and mild fibrin exudation attributed to peri-mortem euthanasia. |
| K2A 50 mg/kg | F | Heart, Spleen, Liver, Kidney, Thymus, Duodenum, Jejunum, Ileum, Colon, Uterus, Ovary, Urinary Bladder, Adrenal, and Pancreas and Stomach are normal. The lung had pulmonary hemorrhage and mild fibrin exudation attributed to peri-mortem euthanasia. |
| K2A 50 mg/kg | M | Heart, Spleen, Liver, Kidney, Thymus, Duodenum, Jejunum, Ileum, Colon, Testes, and accessory sex glands are normal. The lung had mild pulmonary hemorrhage and mild fibrin exudation attributed to peri-mortem euthanasia. Some of the Renal Tubular epithelium were observed to have produced a proteinaceous eosinophilic intra-luminal exudate that is considered normal in male mice; this was a consistent finding in all of the males in this study. No inflammation was observed in or around the Renal Tubules. |
| K2A 50 mg/kg | M | Heart, Spleen, Kidney, Thymus, Duodenum, Jejunum, Ileum, Colon, Stomach, Pancreas and Testes are normal. The lung had mild pulmonary hemorrhage and mild fibrin exudation attributed to peri-mortem euthanasia. Some of the Renal Tubular epithelium were observed to have produced a proteinaceous eosinophilic intra-luminal exudate that is considered normal in male mice; this was a consistent finding in all of the males in this study. No inflammation was observed in or around the Renal Tubules. The liver was not present in the slides prepared. |
| K2A 50 mg/kg | M | Heart, Spleen, Liver, Kidney, Thymus, Duodenum, Jejunum, Ileum, Colon, Stomach, Pancreas and Testes are normal. The lung had mild pulmonary hemorrhage and mild fibrin exudation attributed to peri-mortem euthanasia. Some of the Renal Tubular epithelium were observed to have produced a proteinaceous eosinophilic intra-luminal exudate that is considered normal in male mice; this was a consistent finding in all of the males in this study. No inflammation was observed in or around the Renal Tubules. |
| R13A 50 mg/kg | F | Heart, Spleen, Liver, Kidney, Thymus, Duodenum, Jejunum, Ileum, Colon, Uterus, Cervix, Ovary, and Uterus are normal. The lung was observed to have severe pulmonary hemorrhage and collapse with mild fibrin exudation attributed to peri-mortem euthanasia. There was a suppurative vaginitis present. |
| R13A 50 mg/kg | F | Heart, Spleen, Liver, Kidney, Thymus, Duodenum, Jejunum, Ileum, Colon, Uterus, Ovary, and Cervix are normal. The lung had severe pulmonary hemorrhage and mild fibrin exudation attributed to peri-mortem euthanasia. The vagina and stomach were not present in the cut sections. |
| R13A 50 mg/kg | F | Heart, Spleen, Liver, Kidney, Thymus, Duodenum, Jejunum, Ileum, Colon, Stomach, Urinary bladder, Vagina, and Ovary are normal. The lung had severe pulmonary hemorrhage and mild fibrin exudation attributed to peri-mortem euthanasia. The Uterus was dilated and had scant keratinizing cells and a few apoptotic epithelial cells. The Colon was not present in the cut sections. |
| R13A 50 mg/kg | M | Heart, Spleen, Liver, Kidney, Thymus, Duodenum, Jejunum, Ileum, Colon, Stomach, Pancreas and Testes are normal. The lung had mild pulmonary hemorrhage and mild fibrin exudation attributed to peri-mortem euthanasia. Some of the Renal Tubular epithelium were observed to have produced a proteinaceous eosinophilic intra-luminal exudate that is considered normal in male mice; this was a consistent finding in all of the males in this study. No inflammation was observed in or around the Renal Tubules. The Kidney and Thymus were not present in the cut sections. |
| R13A 50 mg/kg | M | Heart, Spleen, Liver, Kidney, Thymus, Duodenum, Jejunum, Ileum, Colon, Stomach, Pancreas, and accessory sex glands are normal. The lung had severe pulmonary hemorrhage and mild fibrin exudation attributed to peri-mortem euthanasia. Some of the Renal Tubular epithelium were observed to have produced a proteinaceous eosinophilic intra-luminal exudate that is considered normal in male mice; this was a consistent finding in all of the males in this study. No inflammation was observed in or around the Renal Tubules. The Testes were not present in the cut sections. |
| R13A 50 mg/kg | M | Heart, Spleen, Kidney, Thymus, Duodenum, Jejunum, Ileum, Colon, and Testes are normal. The lung had severe pulmonary hemorrhage and mild fibrin exudation attributed to peri-mortem euthanasia. Some of the Renal Tubular epithelium were observed to have produced a proteinaceous eosinophilic intra-luminal exudate that is considered normal in male mice; this was a consistent finding in all of the males in this study. No inflammation was observed in or around the Renal Tubules. The Liver was observed to have a single small focus of mixed inflammatory cells which was considered to be an incidental finding. |
| Vehicle Control | F | Heart, Spleen, Kidney, Thymus, Duodenum, Jejunum, Ileum, Colon, Thymus, and Ovary are normal. The lung had severe pulmonary hemorrhage and mild fibrin exudation attributed to peri-mortem euthanasia. The Uterus has small, scattered patches of keratinized epithelium. |
| Vehicle Control | F | Heart, Spleen, Kidney, Thymus, Duodenum, Jejunum, Ileum, Colon, Thymus, Urinary bladder, Uterus and Ovary are normal. The lung had severe pulmonary hemorrhage and mild fibrin exudation attributed to peri-mortem euthanasia. |
| Vehicle Control | F | Heart, Spleen, Kidney, Thymus, Duodenum, Jejunum, Ileum, Colon, Thymus, Uterus, Pancreas and Ovary are normal. The lung had severe pulmonary hemorrhage and mild fibrin exudation attributed to peri-mortem euthanasia. There was a mild suppurative vaginitis present. |
| Vehicle Control | M | Heart, Spleen, Kidney, Thymus, Duodenum, Jejunum, Ileum, Colon, Thymus, Stomach and Testes are normal. The lung had severe pulmonary hemorrhage and mild fibrin exudation attributed to peri-mortem euthanasia. The Kidney, Pancreas and Thymus are missing in the cut sections. |
| Vehicle Control | M | Heart, Spleen, Kidney, Thymus, Duodenum, Jejunum, Ileum, Colon, Thymus, Stomach and Testes are normal. The lung had severe pulmonary hemorrhage and mild fibrin exudation attributed to peri-mortem euthanasia. The Pancreas is missing in the cut sections. |
| Vehicle Control | M | Heart, Spleen, Kidney, Thymus, Duodenum, Jejunum, Ileum, Colon, Thymus, Stomach and Testes are normal. The lung had severe pulmonary hemorrhage and mild fibrin exudation attributed to peri-mortem euthanasia. The Stomach, Pancreas and Thymus are missing in the cut sections. |

* – n=6 for K2A, R13A, and vehicle control groups; all groups contained equal numbers of male and female mice

M/F – male and female
